# Granulomatosis With Polyangiitis Complicated by Diffuse Alveolar Hemorrhage, Splenic Infarction and Stroke

**DOI:** 10.7759/cureus.30102

**Published:** 2022-10-09

**Authors:** Ke Zhang, Mohamed Salih Makawi, Omar Saab, Hadya Elshakh, Priyank Trivedi

**Affiliations:** 1 Internal Medicine, NewYork-Presbyterian Queens Hospital, Flushing, USA; 2 Critical Care Medicine, NewYork-Presbyterian Queens Hospital, Flushing, USA

**Keywords:** vasculitis diagnosis, dah, cerebrovascular stroke, splenic infarction, granulomatosis with polyangiitis (gpa)

## Abstract

Granulomatosis with polyangiitis (GPA, formerly Wegener granulomatosis) is a type of small artery necrotizing vasculitis that presents with various organ manifestations and disease severity. The most commonly and severely affected organs include the nasopharynx, lungs, and kidneys. However, it can have atypical presentations and lead to misdiagnosis. Here we present a case report of a patient diagnosed with GPA complicated by diffuse alveolar hemorrhage (DAH), splenic infarctions, and stroke.

## Introduction

Granulomatosis with polyangiitis (GPA) is traditionally considered a disease of ‘small-to-medium sized vessels,’ with a preference for renal and pulmonary sites. Patients typically present with fever, cough, dyspnea, hemoptysis, low-grade proteinuria, and hematuria. It can also cause severe complications such as diffuse alveolar hemorrhage (DAH), splenic infarction, and stroke. Therefore, it is essential to recognize these manifestations and make an appropriate diagnosis early.

## Case presentation

**Figure 1:**
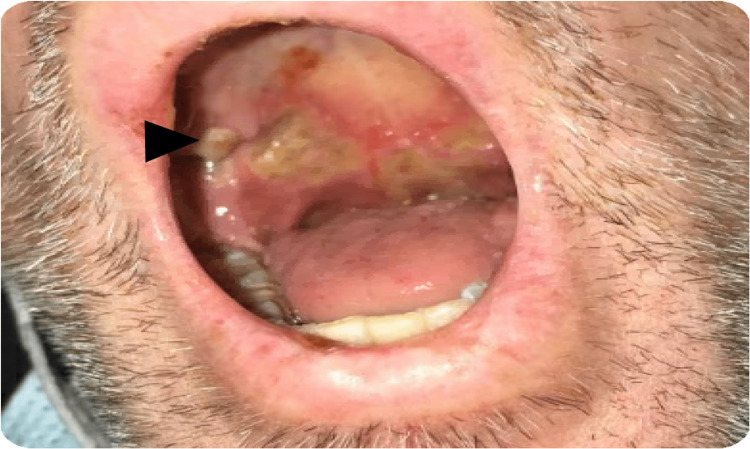
Oral ulcer on palatine (black arrowhead).

**Figure 2:**
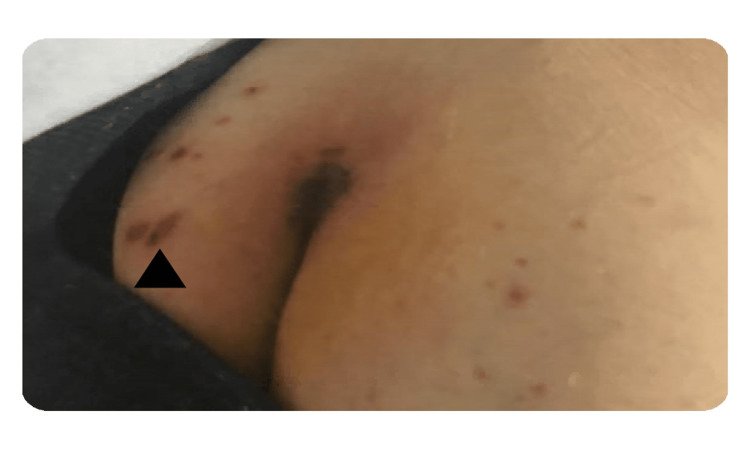
Hyperpigmented rash on the sacral area (black arrowhead).

A 47-year-old male presented to the ED complaining of shortness of breath on exertion, hemoptysis for two weeks, and weight loss of 20 pounds for the past three months, accompanied by loss of appetite. His medical history was unremarkable, and he denied a family history of cancers or rheumatologic disease. However, his social history was remarkable for previous cigarette smoking, use of marijuana, and chronic alcohol use. Vital signs were significant for tachycardia with a blood pressure of 120/84 and pulse of 119/min, and tachypnea with oxygen saturation of 95% on the nasal cannula. Physical examination was positive for bilateral temporal muscle wasting and oral ulcers (Figure 1), bilateral rales on lung examination, and a purpuric rash on the sacral area (Figure 2).

Laboratory studies were significant for normocytic anemia, leukocytosis, and elevated inflammatory markers (Table 1). Urinalysis was positive for microscopic hematuria and proteinuria. In addition, the patient had three negative sputum samples of acid-fast bacilli to rule out tuberculosis. Tests for antinuclear antibody (ANA), HIV, and COVID-19 polymerase chain reaction (PCR) were also negative.

**Table 1:** Laboratory tests on the day of admission and two months later. WBC keeps elevated because the patient was treated with steroids. ESR: Erythrocyte sedimentation rate; CRP: C-reactive protein; RF: Rheumatoid factor.

Lab tests	Admission day	Two months after the first admission	Reference range
WBC count	22.10	22.98	4.80-10.80 K/uL
Hemoglobin	5.0	8.8	13.3-17.7 g/dL
ESR	> 130	61	0-15 mm
CRP	32.3	1.02	0.00-0.49 mg/dL
RF	57	<10	0.0-14.0 IU/mL
anti-CCP	28	3	0-19 units
anti-Pr3	494	10	0-19 AU/mL
anti-MPO	1	1	0-19 AU/mL
anti-SSA	0	0	0-40 AU/mL
anti-SSB	0	0	0-41 AU/mL
anti-dsDNA	4	Not tested	0-24 IU

**Figure 3:**
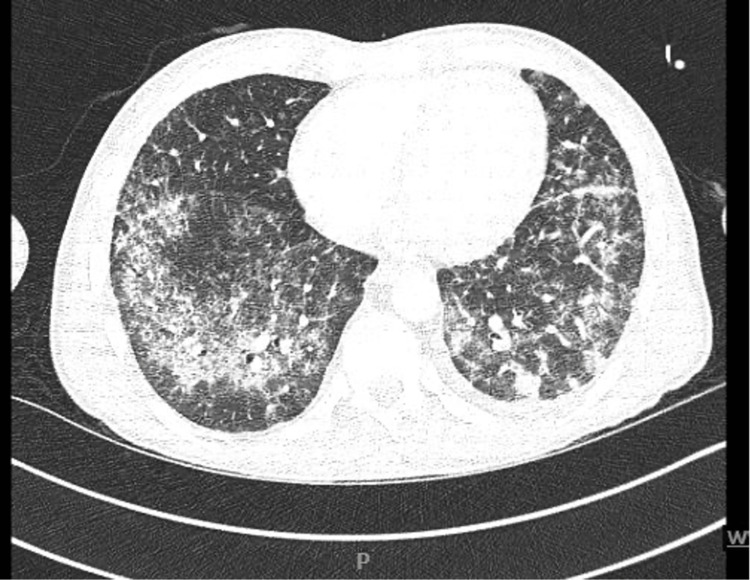
Extensive scattered bilateral airspace opacities confirmed DAH on bronchoscopy. DAH: Diffuse alveolar hemorrhage.

**Figure 4:**
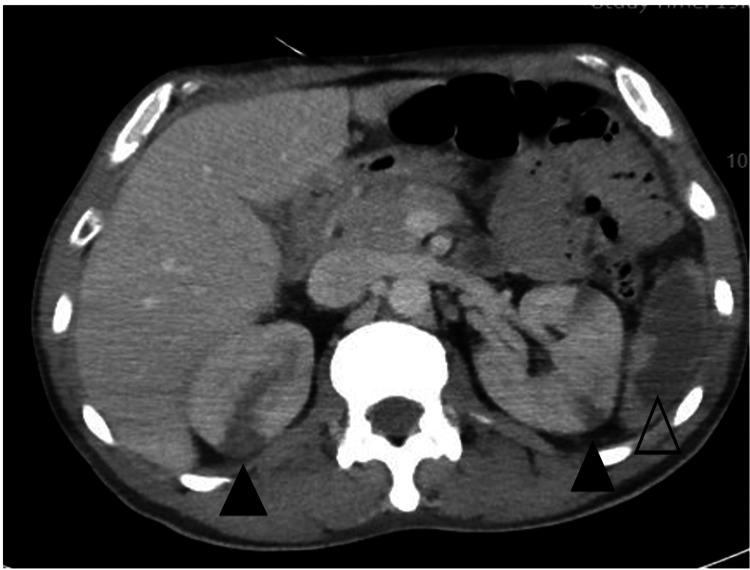
Bilateral renal infarcts (black arrowhead) and extensive splenic infarct (transparent arrowhead) on CT abdomen.

Chest X-ray showed extensive bilateral interstitial opacities and bilaterally basal nodules. CT angiography of the chest was done on day 1 (Figure 3) and showed extensive bilateral airspace opacities, ground glass, and interstitial opacities, most pronounced in the upper lobes bilaterally. Furthermore, CT showed nodularity within the lung bases, most pronounced in the left lung base measuring up to 5 cm. CT scan of the abdomen with contrast was done to investigate the reason for weight loss, and it showed splenic and bilateral renal infarcts (Figure 4).

**Figure 5:**
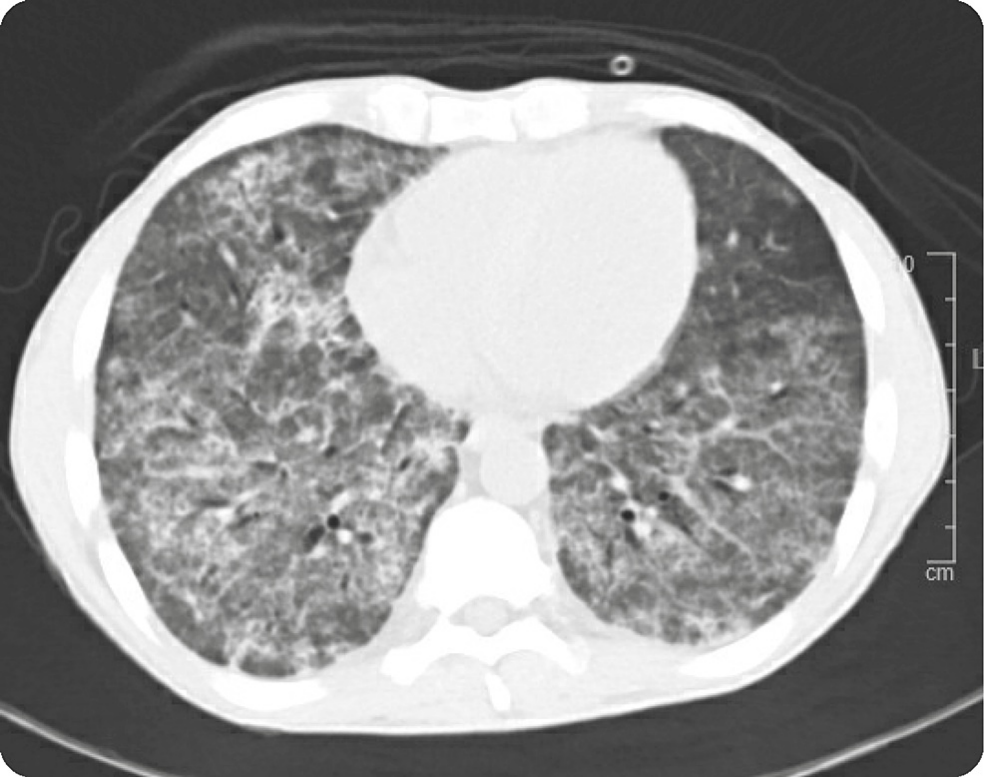
CT chest on day 8, improved nodularity is noticed.

The patient was presumptively diagnosed with community-acquired pneumonia and started with doxycycline and ceftriaxone. However, the next day the patient's hypoxia got worse. Rheumatologic etiology was suspected. Labs were positive for rheumatoid factor (RF), anti-CCP, and anti-Pr3, and negative for anti-dsDNA, anti-MPO, anti-SSA, and anti-SSB (Table 1). We established a diagnosis of GPA based on the positive anti-Pr3 antibody. The patient proceeded with bronchoscopy and bronchoalveolar lavage, which showed DAH. Cytology showed ​​reactive respiratory cells and hemosiderin-laden macrophages in a background of inflammatory cells and fibrin, which are consistent with inflammatory pneumonitis. The patient responded well with methylprednisolone 1g for three days, followed by rituximab 375 mg/m2 weekly. A repeated CT scan on day 8 of hospitalization showed improved pulmonary nodular opacities (Figure 5). He was then discharged and followed up by the rheumatologist. The patient was readmitted about 40 days after discharge due to a severe thrombotic stroke at the right middle cerebral artery. He was treated with a tissue plasminogen activator and thrombectomy and discharged after 10 days to home after being stabilized. There were no respiratory symptoms, abdominal pain, fever, joint pain, or rash.

## Discussion

GPA can be complicated by renal infarction [1-3], and patients with active disease demonstrate altered indices of coagulation and fibrinolysis. This will lead to a hypercoagulable state that increases venous thromboembolic risk, which does not differ between MPO and PR3 vasculitis [4]. Such prothrombotic complications can be seen in patients who have previously achieved remission following immunosuppressant therapy [5]. DAH is also a well-recognized life-threatening complication of ANCA-associated vasculitis (AAV). It can present with a triad of hemoptysis, pulmonary infiltrates, and anemia [6]. In a retrospective review, one-third (8/24) of patients with DAH were found to have AAV [7]. In different studies, the incidence of DAH in AAV is between 8% and 36% [8-10].

Stroke is another complication of GPA. In a retrospective study, the incidence of developing ischemic stroke at 10 years is 11%, four times more than in the general population [11]. Accelerated atherosclerosis has been an emerging pathogenic complication of AAV in recent years. Studies have shown that increased cytokines production, especially T-Helper-17 and T-Helper-1, and lipid accumulation, proceeding to arterial inflammation, is associated with atherosclerosis [12,13]. Increased carotid intima-media thickness is also a reason for ischemic stroke in AAV patients [14]. Studies have found that administering immunosuppressive agents and statins was beneficial in primary stroke prevention [15]. Still, in this study, antiplatelet agents (aspirin, clopidogrel) were not found to have protective effects. However, this study was limited to patients in Korea, and further research on stroke prevention in AAV patients is required.

Splenic infarction is an underestimated manifestation of AAV. The spleen is predisposed to infarction due to occlusive infarction of distal splenic arteries and arterioles and end vessels lacking collaterals [16]. Clinically apparent splenic infarction appears to be a rarer phenomenon with few reported cases but typically presents with left-sided abdominal pain. The patient did not have any abdominal symptoms in this case report, but the CT abdomen with contrast showed splenic infarction. It is not a common finding in AAV in the literature, but it is most likely found in patients with GPA. It can be asymptomatic and probably underestimated, but when present, it can help differentiate microscopic polyangiitis from other AAVs. Though the prevalence and specificity of splenic infarction in GPA are not established, the existing articles point out that splenic infarction is indicative of GPA. One study found that splenic pathologies were observed in 19 patients (28%), of whom seven (37%) had splenic infarction [17]. There are only a few cases that report finding splenic infarction in patients with microscopic polyangiitis (MPA) and eosinophilic GPA [18,19]. As splenic infarction is almost only present in PR3-ANCA positive vasculitis, this helps clinicians differentiate between MPA and GPA. Therefore, physicians should consider GPA in patients with splenic infarctions suspected of having vasculitis [20]. Splenic infarction also should not be ignored in GPA patients as it has been demonstrated that patients may have severe infarction or auto-splenectomy. Thus, clinicians should consider vaccination for preventable infections, including Neisseria, Streptococcus pneumoniae, Haemophilus, etc. In this case, the CT abdomen was done before GPA was diagnosed, but splenic infarction should not be overlooked when a patient is diagnosed with GPA. Doing an abdominal CT to rule out spleen involvement would be beneficial.

## Conclusions

GPA is a multi-system small vessel vasculitis. It presents a diagnostic challenge given that its various symptoms and manifestations may mimic other diseases, such as infections or malignancies. It is crucial to promptly recognize the presentation of GPA, as prompt induction immunosuppression with high-dose steroids, rituximab, or cyclophosphamide is important for a more favorable chance for remission and reducing mortality. DAH is a life-threatening complication in patients with GPA, which can be demonstrated by imaging. Prompt identification of GPA as the etiology of DAH can lead to earlier initiation of treatment, demonstrating improved remission rates. Stroke risk increases in patients with GPA, and management with immunosuppressive agents (glucocorticoids, cyclophosphamide, rituximab, azathioprine/mizoribine, and methotrexate) and statins may mitigate the risk. Splenic infarction should be considered in GPA patients, and splenic imaging should be performed in this group of patients to avoid misdiagnosis. Splenic and renal infarcts may be associated with increased morbidity and mortality in this patient population, and more data is required to assess these risks.
